# Self-esteem in Early Adolescence as Predictor of Depressive Symptoms in Late Adolescence and Early Adulthood: The Mediating Role of Motivational and Social Factors

**DOI:** 10.1007/s10964-017-0727-z

**Published:** 2017-08-07

**Authors:** M. Masselink, E. Van Roekel, A. J. Oldehinkel

**Affiliations:** 1 Interdisciplinary Center Psychopathology and Emotion regulation, University of Groningen, University Medical Center Groningen, Groningen, The Netherlands; 2 Department of Developmental Psychology, Tilburg University, Tilburg, The Netherlands

**Keywords:** Self-esteem, Depression, Motivation, Social problems, Avoidance, Social support

## Abstract

Ample research has shown that low self-esteem increases the risk to develop depressive symptoms during adolescence. However, the mechanism underlying this association remains largely unknown, as well as how long adolescents with low self-esteem remain vulnerable to developing depressive symptoms. Insight into this mechanism may not only result in a better theoretical understanding but also provide directions for possible interventions. To address these gaps in knowledge, we investigated whether self-esteem in early adolescence predicted depressive symptoms in late adolescence and early adulthood. Moreover, we investigated a cascading mediational model, in which we focused on factors that are inherently related to self-esteem and the adolescent developmental period: approach and avoidance motivation and the social factors social contact, social problems, and social support. We used data from four waves of the TRAILS study (*N *= 2228, 51% girls): early adolescence (mean age 11 years), middle adolescence (mean age 14 years), late adolescence (mean age 16 years), and early adulthood (mean age 22 years). Path-analyses showed that low self-esteem is an enduring vulnerability for developing depressive symptoms. Self-esteem in early adolescence predicted depressive symptoms in late adolescence as well as early adulthood. This association was independently mediated by avoidance motivation and social problems, but not by approach motivation. The effect sizes were relatively small, indicating that having low self-esteem is a vulnerability factor, but does not necessarily predispose adolescents to developing depressive symptoms on their way to adulthood. Our study contributes to the understanding of the mechanisms underlying the association between self-esteem and depressive symptoms, and has identified avoidance motivation and social problems as possible targets for intervention.

## Introduction

The prevalence of depression increases sharply from around 2% in early adolescence to around 18% in early adulthood (Hankin et al. 1998; Oldehinkel and Ormel 2015). Many factors contribute to this surge in the experience of depressive symptoms during adolescence (Hankin 2006). Low self-esteem has been suggested to be an important factor that increases vulnerability to depression (Beck 1967; Orth et al. 2016). An impressive amount of research has shown that low self-esteem and depressive symptoms often co-occur among adolescents (e.g., Carbonell et al. 1998; Lee and Hankin 2009; Overholser et al. 1995; Sowislo and Orth 2013). Longitudinal studies suggest that the direction of the association between self-esteem and depressive symptoms is predominantly from self-esteem to depressive symptoms rather than the other way around (Sowislo and Orth 2013). The association holds even after controlling for previous levels of depressive symptoms and Big Five personality traits (Sowislo et al. 2014). Low self-esteem thus seems to be a unique factor that makes adolescents vulnerable to develop depressive symptoms. The association between self-esteem and depressive symptoms is particularly interesting to examine during adolescence, as self-esteem affects many of the developmental challenges adolescents have to deal with, such as identity formation (Erikson 1968) and reshaping social relations (Steinberg and Morris 2001). Exploring the developmental pathway from self-esteem to depressive symptoms can shed light on these processes.

Although previous studies have provided insight into the likely direction of the association between self-esteem and depressive symptoms, the underlying mechanism is far from clear. Insight into this mechanism cannot only increase our understanding of the developmental pathway of how low self-esteem may result in depression, it may also foster the development of interventions. Interventions purely aimed at bolstering (short term) self-esteem have been shown to be notoriously ineffective (Baumeister et al. 2003; Swann et al. 2007). However, targeting not only low self-esteem, but also the broader context of factors influenced by self-esteem as well, may provide leads for more effective interventions. To elucidate how self-esteem relates to depressive symptoms, we tested whether early adolescents experiencing low self-esteem remain vulnerable to develop depressive symptoms over prolonged periods of time, and if so, through which mediators.

Self-esteem levels tend to decrease in early adolescence and increase in later adolescence (Baldwin and Hoffmann 2002), but those who have lower levels of self-esteem than others at one time point are likely to have lower self-esteem than others at the following time point as well (Robins and Trzesniewski 2005). This suggests that self-esteem is a stable and enduring vulnerability. Longitudinal studies have been highly valuable in identifying the likely direction of the association between self-esteem and depressive symptoms (i.e., from self-esteem to depressive symptoms), but to a much lesser extent in identifying the time frame in which adolescents with low self-esteem remain vulnerable to developing depressive symptoms. This is partly due to the relatively short duration of most studies that cover multiple time points, with the duration usually ranging between 2 weeks and 2 years (Sowislo and Orth 2013). Studies covering longer time periods often only investigated cross-lagged effects with the previous time point (e.g., Orth et al. 2008). Exceptions are studies conducted by Trzesniewski and colleagues (2006), who found that low self-esteem between ages 11 and 15 years increased the probability of a Major Depressive Disorder at age 26, and a study by Steiger and colleagues (2014), showing that adolescents with low or declining self-esteem between 12 and 16 years were more likely to show depressive symptoms at age 35. These two studies suggest that low self-esteem is a stable vulnerability factor over many years. However, another longitudinal study over 10 years found that, after controlling for potential confounders, self-esteem at age 15 did not meaningfully predict depressive symptoms at age 25 (Boden et al. 2008). Given the limited and contradicting studies, we replicated these studies by investigating whether self-esteem in early adolescence predicted depressive symptoms in late adolescence and early adulthood.

**Fig. 1:**
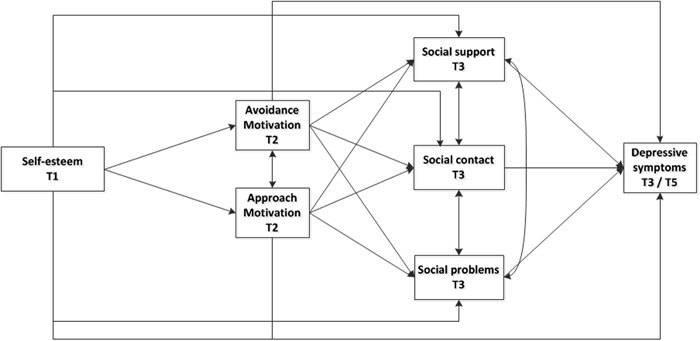
Proposed cascading mediational model from self-esteem to approach and avoidance motivation to social factors and depressive symptoms

The pathway from low self-esteem to depressive symptoms in adolescents is likely to pass through several mediating factors (Kuster et al. 2012; Orth et al. 2016). Identifying those factors facilitates more refined theory building and may ultimately foster the development of focused interventions. In the present research, we looked at two sets of potentially cascading mediators. The first set concerned the question how self-esteem may influence approach and avoidance motivation; the second set was used to explore how self-esteem and approach and avoidance motivation may influence social contact with peers, perceived social support from peers, and social problems (see Fig. 1 for a graphical representation of our proposed model). In the following, we will describe this process in more detail, starting with approach and avoidance motivation.

Self-esteem has received considerable attention in developmental research because self-esteem has a motivational function (Harter and Whitesell 2003), which may affect developmental trajectories. Self-esteem thus not only entails cognitive evaluative aspects of the self, but also motivational ones (Baumeister et al. 1989; Heimpel et al. 2006). Individuals with low self-esteem are characterized by negative views about the self and an avoidance focus to protect the self from possible harm, whereas individuals with high self-esteem are characterized as having an approach motivation to maintain and further enhance self-esteem (Baumeister et al. 1989; Heimpel et al. 2006). These different motivational characterizations for low vs. high self-esteem are similar to what can be expected from activation of the Behavioral inhibition System (BIS) and Behavioral Activation System (BAS) respectively (Carver and White 1994; Gray 1994). The BIS is sensitive to signals of punishment, non-rewards and novelty; and activation of this system is related to avoidance and inhibition of goal pursuit. The BAS, on the other hand, is sensitive to reward, non-punishment and escape from punishment; and activation of this system is related to goal setting, pursuit and maintenance. Research findings have indicated that self-esteem is indeed negatively related to activation of the BIS, and positively related to activation of the BAS (Erdle and Rushton 2010; Kuppens and Van Mechelen 2007; Park 2010). High activation of the BIS and low activation of BAS have also been proposed to relate to depression (Kasch et al. 2002; Shankman and Klein 2003; Gray 1994), and research results are generally consistent with this reasoning (Trew 2011). High levels of BIS are often conceptualized as indicators of avoidance motivation and high levels of BAS as indicators of approach motivation (Elliot and Thrash 2002). In the remainder of this article, we will therefore refer to approach and avoidance motivation. Although approach and avoidance motivation may be directly related to depressive symptoms, they may do so indirectly via social contact, social problems, and perceived social support.

Many of the developmental challenges that adolescents face revolve around their position in their social environment (Steinberg and Morris 2001). These challenges include changing schools, building new social networks, changing relations with family members, adopting an increasingly more adult role over time, and identity formation (Forbes and Dahl 2010; Steinberg and Morris 2001). Peers play a complex role in the lives of adolescents. On the one hand, peers can be sources of interpersonal stress, which has been proposed to be one of the leading causes of depressive symptoms during adolescence (Hankin et al. 2007). On the other hand, adolescents also increasingly rely on their peers, and peers become the most important source of social contact and social support (Levitt et al. 1993; Steinberg and Morris 2001). Not being able to face the social challenges and to fit in with peers may have adverse consequences, through various pathways. First, adolescents who are not able to adopt, maintain and build new social networks may fail to fulfill their basic human need to belong (Baumeister and Leary 1995). A lack of social contact has been related to the experience of depressive symptoms and negative affect (Hopko and Mullane 2008; Lennarz et al. 2016). Second, adolescents may receive insufficient social support to deal with the challenges they are faced with. The importance of social support has been highlighted by several studies, and a lack of perceived social support has been shown to relate to depressive symptoms (Galambos et al. 2004; Lee et al. 2014). Third, for successful integration into new social networks, adolescents have to be socially adjusted. Various forms of social adjustment problems have been associated with depressive symptoms among adolescents (Allen et al. 2006). Social factors thus seem important predictors of depressive symptoms, and are likely to remain so throughout adolescence due to the continuously changing and developing social demands (e.g., developing romantic interests, transition from secondary school to college or university). Compared with adolescents with high self-esteem, adolescents with low self-esteem report a smaller social network (Marshall et al. 2014; but see Stinson et al. 2008), more social problems (Egan and Perry 1998), and lower levels of social support (DuBois et al. 2002; Marshall et al. 2014). Below we will describe how these social factors may be affected by self-esteem and approach and avoidance motivation.

Because approach and avoidance motivations regulate goal setting, the motivational system influences how individuals interact with the world and what activities they engage in. An individual with avoidance motivation may have the goal to avoid rejection by peers. One strategy would be to put extra effort in being liked, but the negative expectations about the own ability to do so that go along with low self-esteem may also lead to another strategy: avoidance of social interaction (Grotevant 1987). An individual with approach motivation, on the other hand, may actively seek out social interactions because it can enhance the feeling of self-worth. We thus expect approach motivation to be positively associated with social contact and avoidance motivation to be negatively associated with social contact.

Over time, approach and avoidance responses may take the form of a reinforcing cycle, and by doing so exert enduring effects on adolescent development. Reactions to certain situations may evolve into social schemas that are used in future situations (Crick and Dodge 1994). When an individual with low self-esteem is successful in avoiding harm to the self by restricting involvement in social interactions, this success is stored in memory and may be retrieved in a later instance, therefore making it more likely that the same strategy will be used. Over time, this can lead to a lack of social skills, as these skills are acquired by trying and learning from previous occasions (Crick and Dodge 1994; Rubin et al. 1998). Avoidance motivation may thus lead to less opportunities to develop the social skills required for successful social interactions. On the opposite, individuals with high approach motivation may have and take more opportunities to work on their social skill development and will therefore experience less social problems.

Strachman and Gable (2006) showed that, compared to people with few social avoidance goals, people with more social avoidance goals tend to have better memory for negative information, are more likely to interpret ambiguous social cues as negative, and are more pessimistic in their evaluations of social actors. On the one hand, individuals with an avoidance motivation may perceive to receive little social support due to their negative expectations and interpretations, on the other hand they may also participate in less social interactions and therefore receive less social support.

There may be gender differences in the associations between self-esteem, the mediators, and depressive symptoms. Starting from early adolescence, girls report more depressive symptoms (Bennik et al. 2014; Hankin et al. 1998), lower self-esteem levels (Fichman et al. 1996), higher levels of avoidance motivation (Jorm et al. 1998), higher levels of perceived social support from friends, and more friends than boys (Cheng and Chan 2004; Rueger et al. 2009). However, associations between self-esteem and depression (Orth et al. 2009; Rieger et al. 2016) and between self-esteem and social support or social contact (Marshall et al. 2014; Stinson et al. 2008) do not seem to differ between boys and girls. Some evidence from research in adolescent and adult samples suggests gender differences in the association between perceived social support and depressive symptoms (Kendler et al. 2005; Rueger et al. 2009). Overall, however, the picture is one of gender differences on the mean level rather than on the level of associations. In our model we thus expected to find similar associations for boys and girls.

## Research Questions

Based on the above-described considerations, we tested a theoretical model (Fig. 1) in which the association between self-esteem and depressive symptoms is partly mediated by approach and avoidance motivation and social factors. More specifically, we tested whether (1) self-esteem in early adolescence predicted depressive symptoms in late adolescence and early adulthood; (2) self-esteem predicted approach and avoidance motivation; (3) approach and avoidance motivation predicted social contact with peers, social problems, and social support from peers; and (4) the social factors served as mediators of the relation between approach and avoidance motivation and depressive symptoms. We also investigated whether the associations in our model were equal across genders.

## Methods

### Sample

The adolescent data came from the first (T1, 10–12 years), second (T2, 12–15 years) and third (T3, 14–18 years) wave and the adult data from the fifth (T5, 21–24 years) wave of the Tracking Adolescents’ Individual Lives Survey (TRAILS). TRAILS is a large prospective cohort study following young adolescents up into adulthood, conducted in the northern part of the Netherlands, with assessment waves 2–3 years apart. The data collection for T1 started in 2001; the data collection for T5 was finished at the end of 2013. Recruitment of participants followed a two stage process. First, demographic information of all adolescents born between October 1, 1989 and September 20, 1991 was obtained from five northern municipalities. Adolescents could only be included if their school was also willing to participate. In total, 135 primary schools were approached to participate in the study, of which 122 agreed to participate. Second, parents and children of those schools were approached to participate in the study, of who both had to give informed consent.

After exclusion of participants who could not participate because of serious health or language problems, 2935 children and their parents were invited for the first measurement wave. Eventually 2230 (76.0%; mean age 11.1 years, *SD* = 0.56; 50.8% girls) adolescents participated in the T1 wave. The response rates for the follow-up waves were 96.4% at T2 (*N* = 2149, 51.0% girls, mean age = 13.65, *SD* = 0.53), 81.4% at T3 (*N* = 1816, 52.3% girls, mean age = 16.27, *SD* = 0.73), and 79.7% at T5 (*N* = 1778, 52.7% girls, mean age = 22.29, *SD* = 0.65). More detailed sample descriptions can be found elsewhere (Oldehinkel et al. 2015). A total of 102 cases had too much missing data across measurement waves to be included in analyses, resulting in a total sample of 2128. Using T-tests we examined whether participants who had missing data on either depressive symptoms at T3 or T5 differed on the other model variables. We only found differences in mean levels between the groups for social contact (mean missing = 15.92, *SD* = 9.25, mean valid = 12.46, *SD* = 8.00, *t* (472.37) = 6.27, *p* < .001) and BIS (mean missing = 2.45, *SD* = 0.53, mean valid = 2.56, *SD* = 0.52, *t* (2088) = 4.50, *p* < .001).

### Measures

At T1 and T2, questionnaires were administered to the participants in their school class under supervision of one or more TRAILS assistants. T3 questionnaires were filled in at school or at home. T5 questionnaires were filled in at home, online or on paper. Descriptive statistics and reliabilities of the measures are reported in Table 1, and zero order correlations in Table 2.

**Table 1:** Descriptive statistics for the independent and dependent variables for boys and girls

		Boys			Girls		
Measure	*α*	*M*	*SD*	*N*	*M*	*SD*	*N*
T1							
Self-esteem	.77	3.38	0.53	1084	3.28	0.55	1124
Depressive symptoms	.77	0.28	0.25	1074	0.3	0.25	1117
T2							
Avoidance motivation (BIS)	.68	2.37	0.49	1016	2.66	0.52	1074
Approach motivation (BAS)	.76	2.9	0.41	1017	2.86	0.42	1074
T3							
Social support	n/a	4.29	0.95	675	4.78	0.49	820
Social contact	n/a	12.86	8.23	758	13.44	8.52	874
Social problems	.76	0.15	0.20	711	0.15	0.21	801
Depressive symptoms	.78	0.22	0.22	777	0.36	0.3	884
T5							
Depressive symptoms	.85	0.24	0.27	653	0.37	0.33	845

*Note*. *α* Cronbach’s alpha reliability coefficient

**Table 2:** Zero order correlations

Measures	1	2	3	4	5	6	7	8
1 Self-esteem T1	–							
2 Depressive symptoms T1	−0.45***	–						
3 Depressive symptoms T3	−0.27***	0.36***	–					
4 Depressive symptoms T5	−0.17***	0.26***	0.48***	–				
5 Approach motivation T2	**−**0.02	0.10***	0.07**	0.06*	–			
6 Avoidance motivation T2	−0.18***	0.23***	0.27***	0.23***	0.18***	–		
7 Social contact T3	0.01	**−**0.02	0.04	0.01	0.08**	−0.10***	–	
8 Social support T3	**−**0.02	0.01	0.07**	0.02	0.02	0.08**	0.19***	–
9 Social problems T3	−0.19***	0.16***	0.26***	0.22***	0.02	0.12***	−0.06*	**−**0.03

*p < .05, **p < .01, ***p < .001

#### Self-esteem

Self-esteem was assessed at T1 with an adjusted version of the 36-items Self-Perception Profile for Children (SPPC) (Harter 1982). This measure has been validated for use in a sample of Dutch school children (Muris et al. 2003). We used the 6-item scale to assess global self-esteem. Instead of the original format (Harter 1982) in which respondents had to decide to which of two descriptions they were most alike, we used a format akin to the one developed by Wichstrøm (1995). In this format, single statements about “some kids” (e.g., “Some kids are satisfied with themselves”) were listed, to which adolescents answered on a 4-point scale ranging from “I do not resemble those children at all” to “I precisely resemble those children”.

#### Approach and avoidance motivation

Approach and avoidance motivation were measured with the 20-item Behavioral Inhibition Scale (BIS) and Behavioral Activation Scale (BAS; Carver and White 1994). This measure was originally developed for adults, but has been shown to be suitable for use in an adolescent population as well (Cooper et al. 2007). Answers were given on a 4-point scale ranging from “very not true” to “very true”. An example BIS item is “I worry about making mistakes”. The BAS measure consists of three subscales (reward responsiveness, drive and fun seeking), which can be combined into one BAS-scale (Jorm et al. 1998). An example of a BAS item is “I go out of my way to get things I want”.

#### Depressive symptoms

Depressive symptoms were assessed at T1 and T3 with 13 items of the DSM-IV based Affective Problems scale of the Youth Self-Report (YSR) questionnaire and at T5 with 14 items of the age-adjusted DSM-IV based Depressive Problems scale of the Adult Self-Report (ASR) questionnaire (Achenbach et al. 2003; Achenbach and Rescorla 2001). Scores of the Affective Problems scale of the YSR have been shown to be strongly related to actual depression diagnosis in a Dutch sample of children, supporting its validity (Ferdinand 2008). All questions of the YSR and ASR were answered on a 3-point scale ranging from “not at all” to “clearly/often”, and concerned the past 6 months. An example item of the YSR/ASR is “I am unhappy, sad, or depressed”.

#### Social problems

To mitigate shared method variance and bias in reporting, social problems were assessed at T3 by one of the parents. We used the 11-item social problems scale of the Child Behavior Checklist (CBCL; Achenbach et al. 2003; Achenbach and Rescorla 2001). Response options were similar to the depressive symptoms measure. An example item is “Doesn’t get along with other kids”.

#### Social contact

Social contact with peers was measured at T3 with items designed by TRAILS about how many hours per week adolescents spent with friends at their home, at the homes of their friends, with their friends outdoors, and going out during the week and weekend. Scores on these items were summed to form the social contact variable.

#### Social support

Perceived social support from peers was measured at T3 as part of the Event History Calendar (EHC), a method to retrospectively obtain data about life events and activities, for the TRAILS study developed into a semi-structured interview of around 45 minutes. Responses on a EHC have been found to correlate highly with questionnaire responses, and proposed to be of superior quality (Belli et al. 2001). During this interview, participants were asked to indicate on a 5-point scale from “never” to “always”, for each of a maximum of seven friends, “Does [name friend] help you when you are having a hard time”. The social support score used in the analyses reflects the highest indicated social support score received from one or more friends (e.g., when someone received a score of 3 and a score of 5, we used the latter). We used the highest received score because adolescents may rely on social support from only some of their friends, not necessarily all of them. As long as sufficient support is received from some friends, support from other friends may be irrelevant.

### Statistical Analyses

All associations between self-esteem and depressive symptoms were investigated using the program Mplus 7.4 (Muthén and Muthén 1998–2015). Missing data were handled using a Maximum Likelihood estimator with robust standard errors to account for non-normality of the variables (MLR).

We first examined the relation between self-esteem and depressive symptoms at T3 and T5 without mediators. Using path analysis, we subsequently expanded the models by including the mediators, BIS and BAS at T2 and social factors at T3. We included paths from self-esteem to all variables in the model to test for both direct and indirect effects. For similar reasons we included direct paths from BIS and BAS to depressive symptoms. Path analysis provides a way to test for direct effects between variables as well as indirect effects. Depressive symptoms at T1 was included as control variable by including paths to all other variables in the model. All effects reported represent standardized coefficients. Due to the fact that we wanted to test for both direct and indirect effects, and control for the influence of depressive symptoms at T1 on all other variables, we had a saturated model. This means that goodness of fit indicators could not be used as indicators of model fit. However, we could test for model fit in multiple group analyses where we constrained associations to be equal for boys and girls. Goodness-of-fit indices included the Chi-square, Comparative Fit Index (CFI), Root Mean Square Error of Approximation (RMSEA), and the Standardized Root Mean Square Residual (SRMR). As the significance level of the Chi-square is highly dependent on the sample size, model evaluations were based on the CFI, RMSEA and SRMR. Models with CFI values > .90 were considered to have acceptable fit and models with a CFI > .95 good fit, RMSEA and SRMR values < .08 indicated acceptable fit and <.05 good fit (Hu and Bentler 1998).

The many paths that had to be estimated in our model had the inherent risk of making Type 1 errors. To mitigate this risk, we applied the False Discovery Rate method (Benajmini and Hochberg 1995). This method takes into account the proportion of significant results of the total number of tests that are performed; a low proportion of significant associations results in a stricter correction than a high proportion of significant results. To calculate the FDR derived significance threshold, an alpha level (.05) is chosen, and the p-values of the performed tests are ranked from low to high. For each ranked test, an FDR threshold is calculated with:

$${\rm{FDR}}\,{\rm{derived}}\,{\rm{significance}}\,{\rm{threshold}} = \frac{{{\rm{0}}{\rm{.05}}}}{{{\rm{number}}\,{\rm{of}}\,{\rm{tests/ranking}}}}$$

The lowest ranked significant p-value which has a p-value below its FDR threshold is used as a cut-off. All ranked p-values above this cut-off are determined to remain significant, all ranked p-values below are labeled insignificant.

## Results

### Self-esteem T1 and Depressive Symptoms T3

**Fig. 2:**
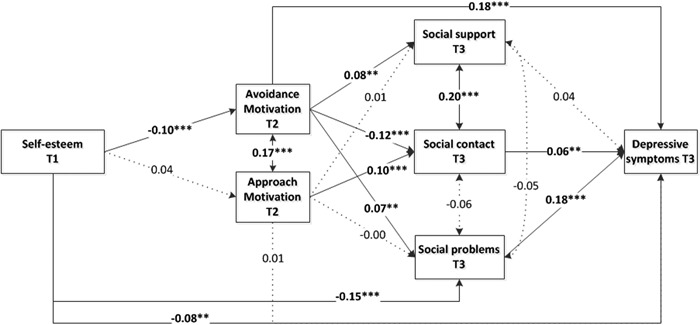
Model with standardized regression coefficients indicating associations between self-esteem and depressive symptoms (T3) with the mediators approach and avoidance motivation and social factors. Non-significant direct paths from self-esteem tot the social factors and associations from the control variable depressive symptoms at T1 are not depicted. *p < .05, **p < .01, ***p < .001

Self-esteem at T1 was significantly related to depressive symptoms at T3 (*β* = −.13, *p* < .001), while controlling for depressive symptoms at T1 (*β* = .30, *p* < .001). The results of the subsequently tested model are presented in Fig. 2. For clarity reasons we did not depict the insignificant direct associations between self-esteem and the social factors, or associations with the control variable. Self-esteem predicted avoidance motivation, but not approach motivation. Avoidance motivation directly predicted depressive symptoms. As expected, avoidance motivation predicted more social problems and less social contact. Surprisingly, avoidance motivation was also related to more social support. Approach motivation predicted more social contact. Social problems were related to more depressive symptoms, and surprisingly, we also found a positive association between social contact and depressive symptoms. Social support was not related to depressive symptoms. The only direct association from self-esteem to the social factors was with social problems. The direct associations with perceived social support and social contact were not significant. In total, 28 correlations and paths over time were tested in this model. All reported associations remained significant after applying the FDR correction which resulted in an adjusted significance threshold of .030.

In a next step, we looked at whether the significant associations from self-esteem and approach and avoidance motivation to depressive symptoms also indicated significant indirect effects. After correcting for the seven tested indirect effects (adjusted significance threshold remained .05), all the tested indirect paths were significant, except the path from self-esteem through avoidance motivation and social contact. The largest indirect path went from self-esteem tot social problems to depressive symptoms (*β* = −.03). The indirect cascading path from self-esteem through avoidance motivation and social problems was *β* = −.001. The total indirect effect was *β* = −.05, *p* < .01 and the total effect of self-esteem on depressive symptoms was *β* = −.13, *p* < .01.

We next tested a model where we included gender in the model as a grouping variable and constrained all paths to be equal for boys and girls. This model had excellent fit (*χ* ^2^ = (28, *N* = 2227) = 37.72, *p* = 0.10, RMSEA = .018, CFI = .986, SRMR = 0.029). This model showed very similar coefficients to the model without gender included as grouping variable, with one exception. For both boys and girls, it was approach motivation that was associated with perceived social support (boys *β* = .04, girls *β* = .08, *p* < .05), not avoidance motivation (boys *β* = −.02, girls *β* = −.03, *p* = .38). This is an indication that the effect from avoidance motivation to perceived social support in the model without gender is an artefact, caused by the so-called Simpson effect, or reversal paradox (Kievit et al. 2013). That is, when two subgroups (i.e., boys and girls) have different mean scores on a variable, combining the data may represent distorted and even reversed overall associations between variables.

### Self-esteem T1 and Depressive Symptoms T5

Self-esteem at T1 was significantly associated with depressive symptoms at T5 (*β* = −.08, *p *<* .01*) when controlling for depressive symptoms at T1 (*β* = .23, *p *< .001). We subsequently tested the same mediational model as before but replaced depressive symptoms at T3 for depressive symptoms at T5. The results showed that self-esteem had no direct effect on depressive symptoms at T5. Social contact at T3 was also not significantly related to depressive symptoms anymore. Avoidance motivation (*β* = .16, *p* < .001) and social problems (*β* = .17, *p *< .001) remained significantly associated with depressive symptoms. Applying the FDR correction did not render any of the reported paths in the model insignificant (28 tests, adjusted significance threshold 0.025). We subsequently looked whether the significant associations from self-esteem and avoidance motivation to depressive symptoms also indicated significant indirect effects. All these paths were indeed significant (4 tests, adjusted significance threshold remained 0.05). The cascading path from self-esteem through avoidance motivation and social problems was again *β* = −.001. The total indirect effect was *β* = −.04, *p* < .01, and the total effect of self-esteem on depressive symptoms at T5 was *β* = −.08, *p* = .01.

In our last model, we entered gender as grouping variable and constrained associations to be equal for boys and girls, which resulted in excellent model fit (*χ* ^2^ = (28, *N* = 2228) = 30.20, *p* = 0.35, RMSEA = .008, CFI = .996, SRMR = 0.027). This model again showed very similar coefficients to the model without gender included as grouping variable.

### Alternate Models Considered

The models presented in this article came about after receiving valuable feedback from reviewers on previously tested models. Changes included incorporating associations between contemporaneous associations and adding direct effects from self-esteem to the social factors. In addition, we removed childhood stress, which was included as potential confounder. There are likely many other possible confounders, and we had no reason to believe that childhood stress was particularly important. Therefore, we decided against including an arbitrarily chosen confounder. We acknowledge the ever present risk of confounders underlying certain associations, without pretending that we adequately dealt with this by including one possible confounder. Importantly, these changes to the model did not alter our conclusions.

The models included a perceived social support variable, which constituted the maximum perceived social support score from up to seven friends. To check whether this operationalization affected the results, we reran the models with the mean perceived social support included in the model instead of the maximum score. The results were identical to the model with the maximum social support variable.

## Discussion

The prevalence of depression increases sharply during adolescence (Hankin et al. 1998). Identification of possible vulnerability factors that predict the development of depressive symptoms is therefore much needed. Many studies have already shown that self-esteem and depressive symptoms are related among adolescents, and longitudinal studies have indicated that the association between self-esteem and depressive symptoms most likely runs predominantly from self-esteem to depressive symptoms (Sowislo and Orth 2013). Self-esteem is thus proposed to be an important vulnerability factor and may be particularly relevant to study in adolescents, because of the important role it plays in social development during adolescence (Steinberg and Morris 2001). However, due to the limited time span of most longitudinal studies, little is known about the duration in which adolescents with low self-esteem remain vulnerable to develop depressive symptoms. Moreover, there is a lack of insight in how low self-esteem may lead to depressive symptoms, because only few studies investigated mediators of the association between self-esteem and depressive symptoms (cf. Kuster et al. 2012). Identification of mediators is not only important for theoretical understanding, it also provides directions for interventions. To address these limitations, we investigated whether young adolescents with low self-esteem remained vulnerable to develop depressive symptoms during late adolescence and early adulthood. In addition, we investigated a cascading mediational model in which the association between self-esteem and depressive symptoms was hypothesized to be mediated by approach and avoidance motivation and social factors.

Our study revealed that self-esteem in early adolescence (mean age 11 years) was directly and indirectly associated with change in depressive symptoms in late adolescence (mean age 16 years) and only indirectly to change in depressive symptoms in early adulthood (mean age 22 years). Our findings concur with two other longitudinal studies (Steiger et al. 2014; Trzesniewski et al. 2006) in which negative effects of low self-esteem on depressive symptoms up to two decades later were found, although our effects were weaker. This suggests that low self-esteem can be a stable vulnerability factor that makes adolescents vulnerable to develop depressive symptoms throughout their way to adulthood. We controlled for earlier levels of depressive symptoms, indicating that self-esteem is a unique predictor of depressive symptoms over time. This goes against some researchers who state that global self-esteem is inseparable from depression (Watson et al. 2002). However, although not unlike several other studies (e.g., Orth et al. 2014), the total effect of self-esteem on depressive symptoms was relatively small. This suggests that, although low self-esteem is a vulnerability to develop depressive symptoms over time, having low self-esteem in early adolescence does not necessarily predispose the individual to develop depressive symptoms during development into adulthood. Still, the development of depressive symptoms is likely not predicted by one or two major factors, but rather by a multitude of factors (Hankin 2006). The fact that self-esteem is a stable predictor of depressive symptoms makes it worthwhile to further investigate the nature of this association.

Our main goal was to examine the mechanism that makes people with low self-esteem vulnerable to develop depressive symptoms. We did so by examining whether approach and avoidance motivation and subsequently social factors, mediated the association between self-esteem and depressive symptoms. Consistent with commonly described characteristics of individuals with low self-esteem (Baumeister et al. 1989; Sowislo and Orth 2013) and previous research (Heimpel et al. 2006), early adolescents with low self-esteem reported more avoidance motivation, suggesting that they seek to avoid possible harmful experiences in order to protect the self from further harm. Moreover, our results support theories of behavioral approach and avoidance motivation (Kasch et al. 2002; Shankman and Klein 2003; Gray 1994) in that avoidance motivation was directly and indirectly related to depressive symptoms in late adolescence and early adulthood. An explanatory process is that high avoidance motivation associated with low self-esteem may result not only in desired avoidance of harm, but also in missing out on possible rewarding instances. This may negatively skew the balance between experienced negative and positive events, leading to more depressive symptoms.

In contrast to the results found for avoidance motivation, self-esteem was not related to approach motivation. This was surprising given evidence indicating that high self-esteem is characterized by sensitivity to rewards and motivation to attain rewards (Erdle and Rushton 2010; Kuppens and Van Mechelen 2007; Park 2010). A potential explanation for these contrasting results could be that approach motivation was assessed regarding events that form a threat to self-esteem (e.g., a negative evaluation) in two of the above-mentioned studies, but not in ours. It is conceivable that, whereas most people are motivated to attain rewards under normal circumstances, only people with high self-esteem are motivated to do so in situations with a self-threat. Further research is needed to investigate whether the relation between self-esteem and approach motivation is actually moderated by the presence of a self-threat.

Another unexpected result regarding approach motivation was that it had no effect on depressive symptoms. This suggests that, in contrast to high avoidance motivation, low approach motivation does not contribute to the risk to develop depressive symptoms. Possibly, approach motivation is specifically related to anhedonia, one of the two core symptoms of depression (Bijttebier et al. 2009). Only one of the thirteen items of the depressive symptoms scale used in this study measured anhedonia, so the measure may not have been sensitive enough to show a relation with approach motivation. Although there is theoretical (Clark and Watson 1991) and empirical support (Hundt et al. 2007; Kimbrel et al. 2007) for this explanation, further research is needed to explicitly test whether approach and avoidance motivation relate to different aspects of depression (e.g., feeling sad and anhedonia).

In addition to avoidance motivation, social problems were an important mediator of the association between self-esteem and depressive symptoms. Self-esteem in early adolescence was directly, and indirectly via avoidance motivation, associated with social problems in late adolescence. It is important to highlight that social problems were measured by parent report, thus indicating a more or less objective measure of social problems rather than a possibly biased perception by the adolescent. The fact that avoidance motivation predicted social problems is in line with the idea that social skills have to be learned over time (Rubin et al. 1998), and that avoiding social interactions impair this development. Social problems, in turn, were associated with depressive symptoms in late adolescence and early adulthood. Although we found significant indirect effects for the mediating role of social problems through avoidance motivation and directly from self-esteem, the effect size of the effect through avoidance motivation was negligibly small. Social problems thus seem to function as a direct mediator between self-esteem and depressive symptoms, not in a cascading manner through avoidance motivation.

As the social problems measure used involved a lack of social skills (e.g., “Doesn’t get along with other kids”) as well as a negative perception of the environment (e.g., “Not liked by other kids”), there are at least two explanations for the mediating role of social problems. First, because individuals with low self-esteem tend to interpret social interactions more negatively and in a self-depreciatory manner (Strachman and Gable 2006), they may misjudge social situations and therefore fail to respond appropriately, leading to social problems. Feeling unable to cope with the social environment can be very distressing, especially for adolescents, thus leading to depressive symptoms. Second, negative perceptions of the social environment may result in a self-fulfilling prophecy leading to fewer reported positive interactions (Downey et al. 1998; Strachman and Gable 2006). This dearth of positive interactions may cause an unfulfilled need to belong resulting in depressive feelings. Regardless of the explanation, our results show the influence of self-esteem on social problems and stress the importance of positive and successful social interactions during adolescence, a time where strong social bonds are formed, and fitting in is particularly important (Steinberg and Morris 2001). The results thus suggest that interventions may not only be aimed at increasing self-esteem, but also on reducing social problems, for example with social skill training.

For the other social factors, expected associations were not found or in contrast with our expectations. Avoidance motivation was positively instead of negatively associated with social support. However, the constrained models for boys and girls suggested that this is the result of a so-called Simpson effect (Kievit et al. 2013), that is, the effect found in the whole sample reversed within the subgroups (i.e., boys and girls) that made up the sample. In the model with gender included, the association of avoidance motivation with perceived social support was in the expected negative direction but not significant. In this model it was approach motivation that was positively and significantly associated with perceived social support. Social support was not related to depressive symptoms, which contrasts research showing clear beneficial effects of social support with regard to experienced psychological distress and depression (DuBois et al. 2002; Lee et al. 2014). That said, Orth et al. (2014) did not find an effect of social support on depressive symptoms either. An explanation for our null-finding may be that the social support measure only concerned perceived social support from friends, not from other important sources of social support like family members (DuBois et al. 2002; Lee et al. 2014). It is possible that, although peers become the main source of social support during adolescence, support from family members remains pivotal for emotional well-being. On the other hand, Orth and colleagues (2014) did measure social support from multiple sources and still found no effect on depressive symptoms, so more research is needed to identify moderating factors leading to these contrasting findings regarding the role of social support.

Another unexpected finding was that social contact was only associated with depressive symptoms in late adolescence, and positively instead of negatively, albeit weakly. This positive association might reflect a misbalance between social bonding with peers, and bonding with parents. As Deković and Meeus (1997) noted, “High involvement with peers during adolescence could be an indicator of lack of attention and concern at home, rather than an indicator of social competence” (p. 173). Adolescents scoring particularly high on our social contact measures may have been those who rely almost exclusively on social contact with peers. Alternatively, a subgroup with high levels of low quality social contact may have disproportionally contributed to the overall effect with a positive association with depressive symptoms, so masking the expected negative association between social contact and depressive symptoms in the other part of the sample. This remains speculation as well because we do not know the quality of the reported social contact, nor the type of friends.

Concurring with others (Orth et al. 2009; Rieger et al. 2016), the excellent model fit of our models in which associations were constrained to be equal for boys and girls suggest that there are no meaningful gender differences in the associations between self-esteem and depressive symptoms. Thus self-esteem and depressive symptoms seem to be comparably related in boys and girls.

Our study has some clear strengths in comparison with prior research. The large sample provided us with enough power to examine a relatively elaborate model. Our longitudinal design from early adolescence into early adulthood enabled us to investigate the prospective relation of self-esteem on depressive symptoms across an important stage of development. In addition, it enabled us to conduct mediation analyses largely prospectively, providing less biased results than cross-sectional mediation analyses (Maxwell and Cole 2007; Selig and Preacher 2009). We tested several variables previously identified to be related to self-esteem and depressive symptoms in one comprehensive model, and could thus account for influences between those variables. Finally, unlike the common practice in testing path-models, we controlled for multiple testing by applying the False Discovery Rate (FDR) method (Benajmini and Hochberg 1995), which provides a balance between decreasing the risks of Type I errors and losing too much power.

Some limitations have to be mentioned as well. Several of these limitations relate to the fact that the data were not collected specifically for this study, and we thus had to capitalize on what was available. First, self-esteem was only measured at the first measurement wave which precluded us from assessing the stability and change of self-esteem over time. Not only the level of self-esteem (Sowislo et al. 2014), but also patterns of change may be important vulnerability indicators (Kernis et al. 1998; Steiger et al. 2014). Due to the one-time measurement of self-esteem, we were not able to test effects of depressive symptoms on self-esteem and how these may have affected the mediators included in our models. Although less consistently and weaker than vulnerability effects, the opposite so-called scar effects have been found in previous research (Shahar and Davidson 2003; Sowislo and Orth 2013; Steiger et al. 2015), so we cannot rule out that such effects influenced the reported results. Another consequence of this limitation is that we were not able to test whether the social factors affected self-esteem over time. Effects from social factors to self-esteem are predicted by sociometer theory (Leary 2005). Sociometer theory conceptualizes self-esteem as an indication of social bonding. Being rejected by others indicates low relational value and thus low self-esteem. Several studies provide support for this view (Gruenenfelder-Steiger et al. 2016; Srivastava and Beer 2005). Effects from self-esteem to social factors are therefore very likely to be reciprocal. The same applies to associations between approach and avoidance motivation and social factors. In reality the mechanism is thus likely much more complex than we were able to test. Second, we only included approach and avoidance motivation and social factors as mediators. Many more variables are likely to play a role, for example rumination (Kuster et al. 2012), dampening of positive effect (Wood et al. 2003), and dysfunctional coping strategies (Lee et al. 2014). Furthermore, many other mediators may influence the relation of the social factors in our model and depressive symptoms, for example peer acceptance (Sentse et al. 2010) and loneliness (van Roekel et al. 2016; Vanhalst et al. 2012). Third, we were not able to control for the mediators on earlier time points, and the social factors were measured at the same time point as depressive symptoms at T3, which possibly resulted in spuriously inflated estimates (Cole and Maxwell 2003). This means, for example, that we do not know whether the association between self-esteem and avoidance motivation reflects a dynamic association or a stable association, possibly driven by other factors. The association between social contact and depressive symptoms in late adolescence may be an artefact of measuring both measures at the same time. Although the same applies to social problems, this association was much more robust, with an equal association with depressive symptoms at the later time point. Fourth, the social support and social contact measures were created for the purpose of the TRAILS study and are not yet tested for validity. Fifth, the relatively long time periods between measurement waves of 2–3 years enabled us to examine effects over long time periods, but precluded investigation of relations over shorter periods. It is very well possible that other and or stronger effects faded away in our design. Sixth, we relied largely on self-report data, which has the risk of increased shared method variance and bias in the responses. That said, highly subjective concepts as self-esteem and experiencing depressive symptoms are hard or even impossible to assess objectively, making self-report the most suitable way of assessment (Sowislo and Orth 2013). Social problems, our measure with the greatest risk for shared method effects and bias by mood-state was measured with parent-report. Seventh, our main model was saturated, meaning that model fit could not be tested. This means that the predictability of the model is unknown. However, results from our constrained model with gender included gave some confidence in our results. In this constrained model we were able to test for model fit, and the fit was excellent. The coefficients for boys and girls were quite similar to what we found in our overall saturated models, providing confidence in our model estimates. Lastly, our analyses did not differentiate between-person effects from within-person effects, which limits the causal and clinical inferences that can be made from our analyses. It is possible that associations that are found between persons are not found within persons, in fact they may even be opposite (Hamaker et al. 2015; Keijsers 2016). Analyses differentiating between-person effects from within-person effects (see for example Hamaker et al. 2015; Ormel et al. 2002) are required to further explore association between self-esteem and depressive symptoms. Nevertheless, our study identified possible important mediators that could be included in future more sophisticated research designs.

In addition to clarifying several aspects of the mechanism underlying the relation between self-esteem and depressive symptoms, our results raise important questions to be addressed in future research, such as whether self-threats moderate the relation between and self-esteem and approach motivation; whether approach motivation is related to anhedonia instead of negative mood; and arguably most important whether the effects replicate within-persons. Moreover, although we have no reason to believe that the mechanisms we found are only at play among adolescents, the path via social problems may be especially pronounced among adolescents, considering that social challenges are prevalent during adolescence. The path via avoidance motivation may be less dependent on the developmental period. It would therefore be interesting to see whether our model replicates in adult samples.

## Conclusion

Our study contributes to the process of understanding the much researched but little understood association between self-esteem and depressive symptoms in adolescence. We extended existing research by showing that this association is mediated by avoidance motivation and social problems, but not in a cascading manner. This implies that we have identified two relatively independent mediators of the association between self-esteem and depressive symptoms, a behavioral motivational one and a social one. Importantly, as social processes are particularly relevant in adolescent development (Steinberg and Morris 2001), the findings related to the social path may be specific to this age group and not apply to other developmental phases. Our results were reassuring in that, although low self-esteem may make early adolescents more vulnerable to developing depressive symptoms in late adolescence and early adulthood, young adolescents experiencing low self-esteem are certainly not predisposed to experience depressive symptoms during development into adulthood. For those with problematic low self-esteem who do require an intervention, existing therapies may fit very well with their needs. Cognitive Behavioral Therapy (CBT) has been shown to be effective in treating depressive symptoms among adolescents (Asarnow et al. 2001; Crocker et al. 2013) and it can be used to target the negative expectations and habituated avoidance behavior as well as the social problem behavior (Asarnow et al. 2001). Overall, although reported associations were small, we have shown that self-esteem may be an enduring vulnerability to developing depressive symptoms and we have identified possible mechanisms that make young adolescents with low self-esteem vulnerable to developing depressive symptoms later in life.
